# Invasive laser acupuncture targeting muscle: a novel approach to protect dopaminergic neurons and reduce neuroinflammation in a brain of Parkinson’s disease model

**DOI:** 10.1186/s13020-025-01104-2

**Published:** 2025-05-07

**Authors:** Halin Jeon, Ju-Young Oh, Sora Ahn, Mijung Yeom, In Jin Ha, Hong-Seok Son, Seong-Eun Park, Juhan Park, Eugene Huh, In-Yeop Baek, Min-Ho Nam, Changsu Na, Myung Sook Oh, Hi-Joon Park

**Affiliations:** 1 Acupuncture and Meridian Science Research Center (AMSRC), Kyung Hee University, Dongdaemun-gu, Seoul, 02447 Republic of Korea; 2 Department of KHU-KIST Convergence Science Technology, Graduate School, Kyung Hee University, Dongdaemun-gu, Seoul, 02447 Republic of Korea; 3 Korean Medicine Clinical Trial Center, Dongdaemun-gu, Seoul, 02447 Republic of Korea; 4 Department of Biotechnology, College of Life Sciences and Biotechnology, Korea University, Seongbuk-gu, Seoul, 02841 Republic of Korea; 5 Department of Formulae Pharmacology, College of Korean Medicine, Gachon University, Sujeong-gu, Seongnam-si, Gyeonggi-do 13120 Republic of Korea; 6 Brain Science Institute, Korea Institute of Science and Technology (KIST), Seongbuk-gu, Seoul, 02792 Republic of Korea; 7 Department of Acupoint and Meridian, Korean Medical College, Dongshin University, Naju-si, Jeollanam-do 58245 Republic of Korea; 8 Department of Oriental Pharmaceutical Science, College of Pharmacy, Kyung Hee University, Dongdaemun-gu, Seoul, 02447 Republic of Korea; 9 Department of Anatomy and Information Science, College of Korean Medicine, Kyung Hee University, Dongdaemun-gu, Seoul, 02447 Republic of Korea

## Abstract

Parkinson’s disease (PD) affects 1–2% of the global population and presents significant therapeutic challenges. Due to the limitations of existing treatments, there is a pressing need for alternative approaches. This study investigated the effects of invasive laser acupuncture (ILA), which combines acupuncture and photobiomodulation. In this method, optical fibers are inserted into the muscle layers of the acupoint to enhance therapeutic outcomes. Mice with MPTP-induced PD were treated with ILA at 830 nm or 650 nm. Protective effects of nigrostriatal dopaminergic neurons and fibers were assessed by examining TH immunoreactivity in the brain. Neuroinflammation markers in the brain and muscle metabolomic profiles were also analyzed. Comparisons between invasive and non-invasive laser application, as well as the impact of nerve blocking with lidocaine, were also evaluated. ILA at 830 nm (ILA830) significantly improved motor performance and increased the nigrostriatal TH-positive immunoreactivities. It reduced the levels of α-synuclein, apoptotic proteins, and inflammatory cytokines, while increasing anti-inflammatory in the brain. ILA830 also decreased nigrostriatal astrocyte and microglia activation. Muscle metabolomic analysis showed distinct group clustering and significant changes in metabolites like glucose and galactose, correlating with improved motor functions. Invasive laser treatment was more effective than non-invasive, and lidocaine pre-treatment did not block its effects. ILA at 830 nm effectively ameliorates PD symptoms by protecting dopaminergic neurons, and reducing neuroinflammation in the brain. Muscle metabolomic changes by ILA830, such as increased glucose and galactose, correlate with motor improvement. This approach offers a promising strategy for PD treatment, warranting further research to optimize its use in clinical settings.

## Introduction

Parkinson’s disease (PD) is the most common movement disorder and the second most common neurodegenerative disorder after Alzheimer’s disease [1, 2]. It has a significant impact on over 10 million individuals globally, and this figure is increasing at a rapid pace [3]. Patients with PD exhibit gradual loss of dopaminergic neurons in the substantia nigra (SN) and dopamine content in the striatum (ST), which is associated with motor symptoms such as bradykinesia, resting tremor, myotonia, and nonmotor symptoms such as cognitive dysfunction, depression, anxiety, and sleep disorder [4, 5]. At present, levodopa, the most frequently prescribed medication, serves as the cornerstone of PD treatment. It does not cure the disease or halt its progression but merely replenishes the dopamine deficiency in the brain [6]. Furthermore, motor complications, including dyskinesia and bradykinesia, may manifest in 33–54% of patients 3–5 years after levodopa treatment [7]. Hence, there is a pressing necessity to research alternative therapeutics for PD, and numerous approaches are currently under investigation [8].

Acupuncture is an essential component of traditional East Asian medicine, and it remains one of the prominent treatment approaches employed today [9]. Animal studies have shown that acupuncture can protect nigrostriatal dopaminergic neurons in PD model mice [10, 11], and multiple clinical research have confirmed the efficacy of acupuncture in managing PD symptoms [12, 13]. Recently, there have been endeavors to augment the effects of acupuncture by incorporating technology that provides additional physical stimulation to acupuncture points.

Photobiomodulation (PBM) is a therapeutic technique that utilizes light to modulate the physiological processes of the body [14]. It does not rely on vibration or heat to induce a biological reaction, but instead utilizes red to infrared light with a power of 500 mW or lower, mainly within the wavelength range of 600–1100 nm. PBM has been shown to reduce pain and inflammation while promoting cell survival and proliferation [15]. Beyond its local effects, such as enhancing wound healing and alleviating knee arthritis [16], PBM has also demonstrated remote effects, including modulation of brain inflammation when applied to the legs or abdomen in PD models [17]. Furthermore, laser acupuncture (LA), which integrates traditional acupuncture with PBM, has been developed and is now in use [18, 19].

The existing LA involves using a laser to target the skin’s surface at acupuncture points. This technique is non-invasive and consequently painless [19]. However, concerns have been raised about whether the laser can effectively stimulate the target tissues, as it is applied to the skin’s surface and may not reach deeper tissues [20]. To address these issues, invasive LA (ILA) was developed as an advanced form of LA. ILA utilizes laser optical fibers embedded within the needle, allowing the laser light to emanate precisely from the needle’s tip once it is inserted into the target tissue. This design enables accurate targeting of specific anatomical structures, such as the dermis or muscle, by adjusting the needle’s depth, thus providing fine control over the laser’s irradiation location. Consequently, ILA can create a synergistic effect between manual acupuncture and PBM, potentially enhancing therapeutic outcomes. Our team’s research highlights the significant advantages of ILA, demonstrating its efficacy in treating musculoskeletal conditions such as low back pain [21, 22]. Notably, this study is the first to explore the potential of ILA in treating neurodegenerative disorders such as PD, marking a pioneering step in this innovative therapeutic approach.

In this study, our goal was to elucidate the therapeutic potential of ILA in a PD mouse model by identifying specific ILA stimulation parameters, such as wavelength, that are effective in improving behavioral outcomes and protecting nigrostriatal dopaminergic neurons. Additionally, we aimed to uncover the molecular mechanisms in the brain that mediate the neuroprotective effects of ILA. We also investigated the factors influencing the brain’s response to peripheral ILA stimulation. To comprehensively address these objectives, we examined the necessity of muscle stimulation, the role of neural transmission, and, if muscle stimulation proved crucial, the role of metabolomic changes in the muscle in achieving therapeutic effects. This research aims to provide robust preclinical evidence and establish optimal conditions that can inform clinical applications, thereby offering substantial insights into the therapeutic potential of ILA for neurodegenerative disorders like PD.

## Methods

### Animals

Male C57BL/6 J mice, aged 8 weeks and weighing 23–25 g, were obtained from Central Animal Laboratories Inc., Seoul, Korea. The mice were kept together in cages with three to five mice per cage. The temperature was maintained at 23 ± 1 °C with a relative humidity of 60 ± 10%. The mice were exposed to a continuous 12-h light/dark cycle, with the light phase from 8:00 a.m. to 8:00 p.m. and the dark phase from 8:00 p.m. to 8:00 a.m. They were provided with unlimited access to food and water. Prior to the start of the trial, the mice were acclimatized in the same environment for 7 days to mitigate animal distress. The procedures followed the rules set by the National Institutes of Health (NIH) and were authorized by the Institutional Animal Ethical Committee at Kyung Hee University (KHSASP-23-280).

### Induction of subchronic PD model

A daily intraperitoneal injection of MPTP (30 mg/kg per day, Sigma-Aldrich, Missouri, USA; 23007-85-4) diluted in saline was used to induce PD subchronically for 5 days. Control group animals were administered saline injections alone.

### Experimental group allocations

To investigate the impact of ILA on PD, mice were randomly assigned to distinct experimental groups. The first experiment aimed to uncover the optimal wavelength and included the following groups: control (CON), MPTP, 830 nm ILA (ILA830), 650 nm ILA (ILA650), and non-LA (NLA) (each *n* = 6). To study signaling pathways and neuroinflammatory mechanisms, the groups were: CON, MPTP, ILA830, and NLA (each *n* = 5). The experiment focused on finding the optimal biological target involved groups: CON, MPTP, ILA830, LA830, and Lidocaine + ILA830 (each *n* = 6). Lastly, the study examining metabolomic alterations in the muscle consisted of the following groups: CON, MPTP, ILA830, and NLA (each *n* = 6).

### Interventions

ILA treatment was administered 2 h after MPTP administration during the induction period of PD and continued 2 h after the same time point each day throughout the remaining duration of the trial. The acupoint GB34, known for its efficacy in restoring motor capabilities impaired by PD [23–25], was subjected to laser irradiation using a medical equipment called Ellise (manufactured by Wontech Co. Ltd., Daejeon, Republic of Korea). Its primary components are a laser output device, a sterile, disposable, stainless steel acupuncture needle with an external diameter of 0.3 mm, an inner diameter of 0.15 mm, and a length of 30 mm, into which an optical fiber is inserted, and an optical fiber-coupled laser diode (InGaAIP at 650 nm, GaAIAs at 830 nm). The acupoint GB34 is situated in a concave area that is positioned in front of and below the head of the fibula. Before undergoing acupuncture therapy, each mouse was restrained in a securing device. The mice in the acupuncture groups were administered sterilized acupuncture needles, which were inserted bilaterally at the GB34 acupoint to a depth of 3 mm. The laser groups (ILA830 and ILA650) were exposed to laser irradiation for 10 min at 650 or 830 nm. The NLA group was not subjected to any laser irradiation for the same duration. The LA830 group went through non-invasive laser treatment in which laser irradiation was treated on the skin surface of the acupoint. In the Lidocaine + ILA830 group, mice were pre-treated with a nerve blocker lidocaine at the site of ILA 3 min prior to ILA treatment. The mice in the CON and MPTP groups underwent a 10 min immobilization stress using the same fixation apparatus as mice in other groups to replicate the same stress level experienced by the acupuncture groups.

### Rotarod test

An accelerated rotarod test was used to assess motor function, balance, and coordination. Mice were placed in their standard cages and were given a minimum of 30 min to acclimate to the testing environment on the day of the experiment. A 2-min acclimatization session at 2 rpm was conducted the day before the test. When a mouse is placed on a spinning rod, the rotarod test measures how long the mouse can stay balanced as the rotation speed increases over a period of 480 s. After increasing the speed from 3.5 to 35 rpm, the speed reaches its maximum in 5 min. A trial ends for each mouse when it drops off the rod. Automatic timers and falling sensors on the rotating rod were used to score the latency to fall.

### Cylinder test

The cylinder test was used to assess the spontaneous exploration behavior in an unfamiliar setting. Before the experiment, mice were kept for 1 min in a transparent plastic cylinder measuring 12 cm in diameter by 20 cm in height. Following adaption, observers counted the number of times mice touched the cylinder wall while standing on their hindlimbs and leaning their forelimbs on it. The experiment was carried out for 3 min.

### OF test

Utilizing the open field (OF) test, spontaneous locomotor activity of mice was examined. Before each test, all mice were acclimated to the testing environment for 1 h. Each mouse was placed in a white acrylic container with the dimensions of 40 × 40 × 27 cm. In order to monitor the locomotor activities of the mice, a video camera was positioned above the center of the room where the experiment was conducted. The area traveled by the rodents was measured to quantify their locomotor activities, which were tracked by a computerized video-tracking system utilizing the SMART program (Pan Lab Co., Barcelona, Spain). For 5 min, the number of entries to the central zone of the container were recorded.

### Immunohistochemistry

Mice were perfused with phosphate-buffered saline (PBS) and 4% paraformaldehyde (PFA) in 0.2 M phosphate buffer under general anesthesia. Following dehydration, the brains were cryoprotected in 30% sucrose solution and sectioned at an identical thickness of 40 μm with a cryostat microtome (Leica Biosystems, Nussloch, Germany). PBS was used to clean the sections, which were then activated with 1% hydrogen peroxide (H_2_O_2_) for 15 min before being blocked in a solution containing 0.3% bovine serum albumin (BSA) and 3% Triton X-100 for 1 h. After blocking, the sections were activated by rabbit anti-tyrosine hydroxylase (TH; 1:1000 for ST and 1:4000 for SNpc; Santa Cruz Biotechnology, sc-14007). After numerous rinses, the sections were treated for 1 h in PBS with a biotinylated anti-rabbit secondary antibody (both 1:1000; Vector Laboratories Inc., BA-1000). After 1 h of incubation at room temperature with avidin-biotinylated peroxidase complex (Vectastain Elite ABC kit; Vector Laboratories Inc.) in PBS, immunoreactions were detectable with DAB peroxidase substrate kit (Vector Laboratories Inc., SK-4100). The sections were rinsed several times, mounted on slides, dehydrated, and covered with coverslips.

### Immunofluorescence analysis

Under anesthesia, the mice were perfused with paraformaldehyde and PBS in a phosphate buffer (0.2 M). Following dehydration, brain sections measuring 40 µm in thickness were obtained using a freezing microtome manufactured by Leica Biosystems in Nussloch, Germany. The sections underwent a series of steps: PBS cleaning, activation with 1% H_2_O_2_ for 15 min, and blocking for 1 h in a solution containing 3% Triton X-100 and 0.3% BSA. After the blocking process, the sections were stimulated with primary antibodies used are as follow: rabbit anti-TH (1:1000 for ST and 1:3000 for SNpc; Millipore, AB152), chicken anti-TH (1:500; Abcam, ab76442), mouse anti-neuronal nuclei (NeuN; 1:500; Millipore, MAB377), guinea pig anti-glial fibrillary acidic protein (GFAP; 1:500; Synaptic Systems, 17004), chicken anti-GFAP (1:500; Millipore, AB5541), and goat anti-ionized calcium-binding adapter molecule1 (IBA1; 1:500; Abcam, ab5076). The sections were mounted onto slides and covered with coverslips following multiple rinses.

### Imaging and quantification

Immunohistochemical photographs were acquired with a microscope (Olympus Japan Co., Tokyo, Japan; BX53). In order to quantify alterations in the quantity of TH-positive neurons in the SN regions, the proportion of TH-positive neurons in mice treated with MPTP was expressed as a percentage of the CON group. In contrast, the average number of TH-positive neurons in CON mice was normalized to 100%. ImageJ software (NIH) was utilized to quantify the optical density of TH immunofluorescence to image and quantify TH-positive fibers in the ST; the resulting value was expressed as a percentage of optical density.

### Sholl analysis

For Sholl analysis, we have exclusively chosen labeled neurons that do not overlap in order to achieve an unambiguous reconstruction. In order to represent the overall quantity of intersections for each neuron, computations were performed at intervals of 5 μm in the SN and 10 μm in the ST, correspondingly. For each cell, the number of ring intersections was tallied manually. Two glial cells from each mouse were analyzed.

### Western blot

Brain tissue was stored at −80 °C until homogenization in lysis and denatured in a CyQUNT™ cell lysis buffer (Invitrogen, Cralsbad, CA, United States) containing protein phosphatase inhibitors. The homogenate was centrifuged (12,000 rpm for 15 min at 4 °C), and the supernatants were collected. Equal amounts of proteins were separated by 10% SDS–polyacrylamide gels, transferred onto PVDF membranes. The PVDF membranes were blocked with 5% skimmed milk in Tris-buffered saline (150 mM NaCl, 20 mM Tris–HCl, pH 7.4), immune bloted with primary antibodies for 3 days at 4 °C: α-Synuclein (1:500; BD, 610787), NF-κB p65 (1:500; Cell Signaling Technology, #8242), Phospho-Akt (1:1000; Cell Signaling Technology, #4060), Akt (1:1000; Cell Signaling Technology, #4691), Phospho-p38 MAPK (1:500; Cell Signaling Technology, #4511), p38 MAPK (1:500; Cell Signaling Technology, #9212), JNK2 (1:250; Cell Signaling Technology, #9258), Phospho-SAPK/JNK (Thr183/Tyr185) (1:500; Cell Signaling Technology, #4668), Bcl2 (1:500; Cell Signaling Technology, #2764), BAX (1:500; Abcam, ab7977), Anti-β-Actin (1:10,000; Sigma, A1978). After washing, the blots were reacted with peroxidase-conjugated secondary antibodies for 1 h and the protein concentrations were determined by the ECL detection system. Optical density of each band was analyzed using ImageJ.

### Enzyme‐linked immunosorbent assay (ELISA)

The levels of inflammatory factors (IL‐1β, IL‐6, TNF-α, and IL-10) in the ST tissue extracts were measured according to the manufacturer’s protocol. In brief, the striatal tissue was dissected and homogenized using homogenizer in ice‐cold and CyQUNT™ cell lysis buffer (Invitrogen, Cralsbad, CA, United States). The homogenates were centrifuged at 12,000 rpm for 15 min at 4 °C, and supernatants were used for ELISA assay (K0331231, K0331230, K0331213, and K0331186; LABISKOMA, Korea).

### Muscle sample derivatization

Following the freeze drying peroneus longus, extensor digitorum longus, and peroneus digiti quinti muscle samples from mice, 100 μL of a pyridine solution containing *O*-methoxyamine hydrochloride (20 mg/mL) was introduced to each sample. After vigorously mixing each sample using a vortex for 30 s, all samples were placed in a dark environment and incubated at a temperature of 30 °C for a duration of 90 min. The silylation process was carried out by adding 50 μL of *N*-methyl-*N*-trimethylsilyl-trifluoroacetamide. Following vortex-mixing of each, they were subsequently incubated at a temperature of 37 °C for 30 min. An internal standard of ribitol (0.5 mg/mL) was added in a volume of 50 μL. Following centrifugation of the samples at a speed of 12,000 revolutions/min for a duration of 10 min, the liquid portion above the sediment was analyzed. Quality control (QC) samples were created by combining equivalent quantities about 10 μL of each sample before the derivatization procedure. In order to assess the system’s performance and stability, as well as the repeatability of the sample treatment technique, QC samples were evaluated every 10 samples over the entire run.

### Gas chromatography–mass spectrometry (GC–MS) analysis

The samples that underwent derivatization were evaluated using a GC–MS instrument (QP2020, Shimadzu, Kyoto, Japan). The separation of metabolites was performed using a fused silica capillary column (30 m × 0.25 mm ID) coated with Rtx-5MS, manufactured by J&W Scientific in California. One µL sample was injected using an autoinjector with a split ratio of 1:35. The temperature at the front inlet was 230 °C. The temperature of the column was maintained at 80 °C for 2 min without any change, and then increased at a rate of 15 °C/min until it reached 330 °C. It was then kept at this temperature for 6 min without any change. The temperatures of the transfer line and ion source were set at 250 °C and 200 °C, respectively. The process of ionization was accomplished by utilizing a 70 electron volt (eV) electron beam. The rate at which helium gas flowed through the column was 1 mL/min. A total of 20 scans/s were documented within the mass range of 85–500 *m*/*z*. The acquisition of chromatograms and mass spectra was performed using Shimadzu GC solution (Shimadzu, Kyoto, Japan).

### Partial Least Squares Discriminant Analysis (PLS-DA) and metabolites identification

The raw data (CDF) from the GC–MS were converted to the ABF format using ABF Converter software. MS-DIAL ver. 4.9.221218, along with an open-source publicly available EI spectra library, were used for various processes, including raw peak extraction, baseline filtering, baseline calibration, peak alignment, deconvolution, peak identification, and integration of peak height, following established methods [26]. The obtained data were exported to Microsoft Excel (Redmond, WA, USA). The aligned peaks were confirmed in the original chromatograms and positively or tentatively identified using either commercial standard compounds in comparison to the mass spectra and retention time, or using the NIST mass spectral database, in-house library, and GC–MS reference material. PLS-DA, which includes supervised projections to latent structures, was then used to a multivariate analysis using the MetaboAnalyst 6.0 program to differentiate metabolites between the two groups.

### Pathway analysis

Pathway analysis was conducted with the MetaboAnalyst 6.0 software. During the analysis, a list of metabolites that were found to be statistically significant was obtained using Student’s *t* test (*p* value < 0.05). The process involved the mapping of important characteristics onto a mix of metabolic models, specifically the Kyoto Encyclopedia of Genes and Genomes model. The relevance of metabolites enriched inside a certain network was determined by comparing the findings on existing metabolite networks to a null distribution generated from permutations of characteristics.

### Statistical analysis

All data were analyzed and graphed using GraphPad Prism 10 (GraphPad Software Inc., San Diego, CA, USA). In order to compare multiple groups, a one-way analysis of variance (ANOVA) was conducted along with Tukey’s post hoc test. Every value was presented as arithmetical mean ± SEM. A *p* value of less than 0.05 was considered significant, and asterisks denote the significance level: * *p* < 0.05, ** *p* < 0.01, and *** *p* < 0.001.

## Results

### ILA ameliorates parkinsonian motor impairments induced by MPTP

**Fig. 1:**
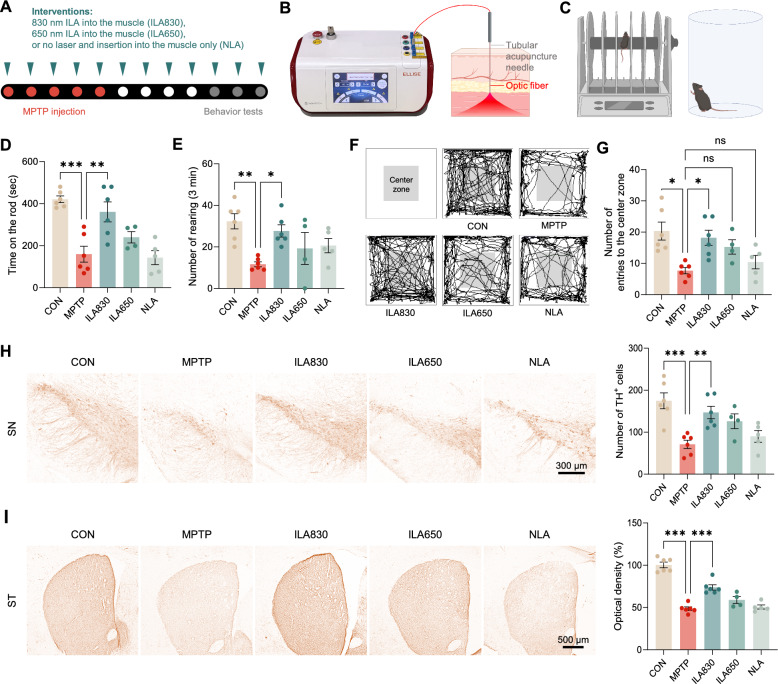
Effects of ILA on motor functions and dopaminergic neuronal death in MPTP-induced PD mice. **A** Experimental design. **B** Image of the novel acupuncture needle integrating with laser technology. **C** Illustrative demonstration of rotarod and cylinder tests. **D** Latency to fall off the rotating rod. **E** Cylinder test results for the number of rearing behaviors. **F** Representative images of OF test. **G** Number of entries into the center zone in the OF test. **H** Representative images and the number of TH-positive cells in the SN. **I** Representative images and optical density of ST. The results were presented as mean ± SEM, * *p* < 0.05, * *p* < 0.01, *** *p* < 0.001

In order to find the optimal wavelength parameter, a novel acupuncture needle combined with laser technology was used to apply two different wavelengths of ILA, 830 nm and 650 nm (Fig. 1A, B). Initially, there were 6 mice in each group for the experiment. However, one mouse from the NLA group and two mice from the ILA650 group died over the course of the trial. To assess the therapeutic effects of ILA in 1-methyl-4-phenyl-1,2,3,6-tetrahydropyridine (MPTP)-induced PD mice, rotarod, cylinder, and OF tests were performed (Fig. 1C). One-way ANOVA showed significant differences between groups in the rotarod test [*F*_4, 22_ = 12.66, *p* < 0.001] (Fig. 1D), cylinder test [*F*_4, 22_ = 5.052, *p* < 0.005] (Fig. 1E), and the number of entries to the center zone [*F*_4, 22_ = 5.858, *p* < 0.05] in OF test (Fig. 1F, G).

In the rotarod test, which measures the duration it takes to fall from a rotating rod, the MPTP group exhibited less than half the performance of the CON group (*p* < 0.001), which had an average time of over 400 s (Fig. 1D). Additionally, the ILA830 group displayed a noteworthy improvement (*p* < 0.01). There was a small rise in the ILA650 group, but it had no statistical significance (*p* = 0.564). The NLA group, which only underwent needling without laser irradiation, showed no difference compared to MPTP group (*p* = 0.997).

The cylinder test is a common behavioral test used to assess motor impairment in experimental models of PD, measuring the number of times a mouse stands up on its hind legs and touches a cylinder with both front paws. The results showed that MPTP treatment significantly decreased the number of times the mouse touched the cylinder (*p* < 0.01), and that laser needling at 830 wavelength restored it (*p* < 0.05). In the ILA650 and NLA groups, there was no significant difference from the MPTP group (Fig. 1E).

Subsequently, in the OF test, we quantified the number of entries to the central zone which reveals the level of anxiety in mice (Fig. 1F, G). It is clear that the MPTP group showed a decrease (*p* < 0.05), but the application of the 830 nm laser needle treatment reversed this reduction (*p* < 0.05).

The behavioral experiments conclusively demonstrate that ILA at a wavelength of 830 nm alleviates motor dysfunction and anxiety in PD model mice, in comparison to 650 nm or NLA. Since the results indicate that ILA with a wavelength of 830 nm is more effective in treating MPTP-induced PD mice compared to 650 nm, the 830 nm laser was selected as the optimal wavelength for this study.

### ILA at 830 nm has neuroprotective effect against MPTP injection

TH is an enzyme that produces levodopa, which is a precursor of dopamine. One way to assess PD models in rodents is by measuring the quantity of TH-positive neurons in the SN after TH staining and quantifying their optical density in the ST. The one-way ANOVA results of the immunohistochemical staining revealed significant differences in dopaminergic neurons in SN [*F*_4, 22_ = 8.164, *p* < 0.001] and dopaminergic fibers in ST [*F*_4, 22_ = 52.91, *p* < 0.001] between the groups (Fig. 1H, I).

The loss of TH-positive dopaminergic neurons in the SN and ST caused by MPTP was significantly recovered by ILA at the wavelength of 830 nm but not without laser irradiation. The cell number of TH-positive dopaminergic neurons decreased in the MPTP group in the SN (*p* < 0.001). The ILA830 group reversed this decline (*p* < 0.01), whereas NLA did not (*p* = 0.9; Fig. 1H). Likewise, in the ST, MPTP injection lowered the intensity of TH-positive fibers (*p* < 0.001), which ILA at 830 nm significantly increased (*p* < 0.001). However, the NLA group did not present the same alterations as ILA 830 treatment (*p* = 0.99; Fig. 1I).

### Neuroprotective effects of ILA treatment through regulation of signaling markers in MPTP-induced PD mice

**Fig. 2:**
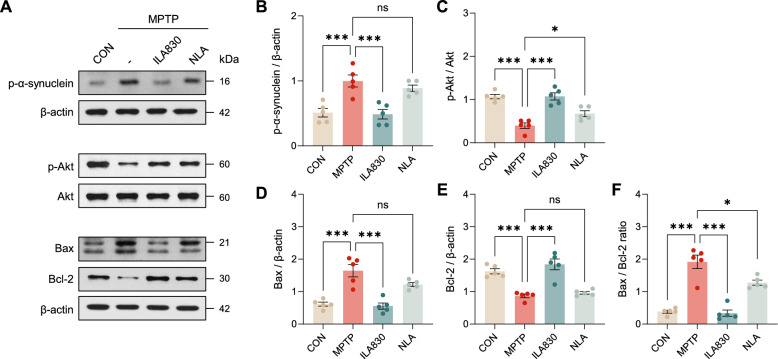
Changes of neuroprotective molecules by ILA830 in the brain. **A** Representative western blot images of α-synuclein, Akt, Bax, Bcl-2, and β-actin in the SN. **B** Western blot analysis of phosphorylated α-synuclein normalized to β-actin in the SN. **C** Western blot analysis of phosphorylated Akt and Akt in the SN. **D** Statistical analysis of the protein expression levels of Bax, normalized to β-actin in the SN. **E** Statistical analysis of the protein expression levels of Bcl-2, normalized to β-actin in the SN. **F** Statistical analysis of the ratio of Bax/Bcl-2 in the SN. Data are shown as the mean ± SEM. * *p* < 0.05, ** *p* < 0.01, *** *p* < 0.001

Α-synuclein is a protein that is neuropathologically related to PD. Thus, we investigated the level of phosphorylation of the protein by western blot (Fig. 2A, B). A one-way ANOVA revealed significant differences across groups [*F*_3, 16_ = 13.22, *p* < 0.001]. Compared to control mice, MPTP group showed significant increase in the level of phosphorylation (*p* < 0.001). ILA successfully reversed this alteration (*p* < 0.001), but no significant effect was observed in the acupuncture group that did not receive laser irradiation (*p* = 0.7) (Fig. 2B).

In order to investigate the signaling mechanisms underlying the neuroprotective effects of ILA, Akt and phosphorylated Akt protein expression levels were evaluated through western blot (Fig. 2A, C). One-way ANOVA showed that the groups were significantly different [*F*_3, 16_ = 23.95, *p* < 0.001]. While MPTP injection alone significantly decreased the phosphorylation of Akt (*p* < 0.001), ILA at 830 nm increased the level of phosphorylation (*p* < 0.001). NLA also increased the phosphorylation level of Akt (*p* < 0.05), although the increase was not as great as ILA. (Fig. 2C).

We investigated the effect of ILA on the pro-apoptotic protein Bax and the anti-apoptotic protein Bcl-2 by western blot analysis (Fig. 2A, D–F). A one-way ANOVA revealed noteworthy variations of Bax levels across the groups [*F*_3, 16_ = 20.68, *p* < 0.001]. MPTP-injected mice showed an increased Bax expression compared to the control mice (*p* < 0.001), which ILA treatment significantly reduced (*p* < 0.001) (Fig. 2D). The results of the one-way ANOVA of the anti-apoptotic protein Bcl-2 indicated statistically significant differences across the groups [*F*_3, 16_ = 23.76, *p* < 0.001]. Bcl-2 was reduced in MPTP-injected mice compared to sham mice (*p* < 0.001), and ILA treatment restored the Bcl-2 expression to the basal levels (*p* < 0.001) (Fig. 2E). Bax/Bcl2 ratio demonstrated a more pronounced difference between the groups (Fig. 2F), with the one-way ANOVA results supporting its significance [*F*_3, 16_ = 37.85, *p* < 0.001]. Taken together, the results demonstrated that ILA attenuates the degree of apoptosis.

### ILA treatment mitigates neuroinflammation by modulating pro- and anti-inflammatory cytokine levels in MPTP-induced PD mice

**Fig. 3:**
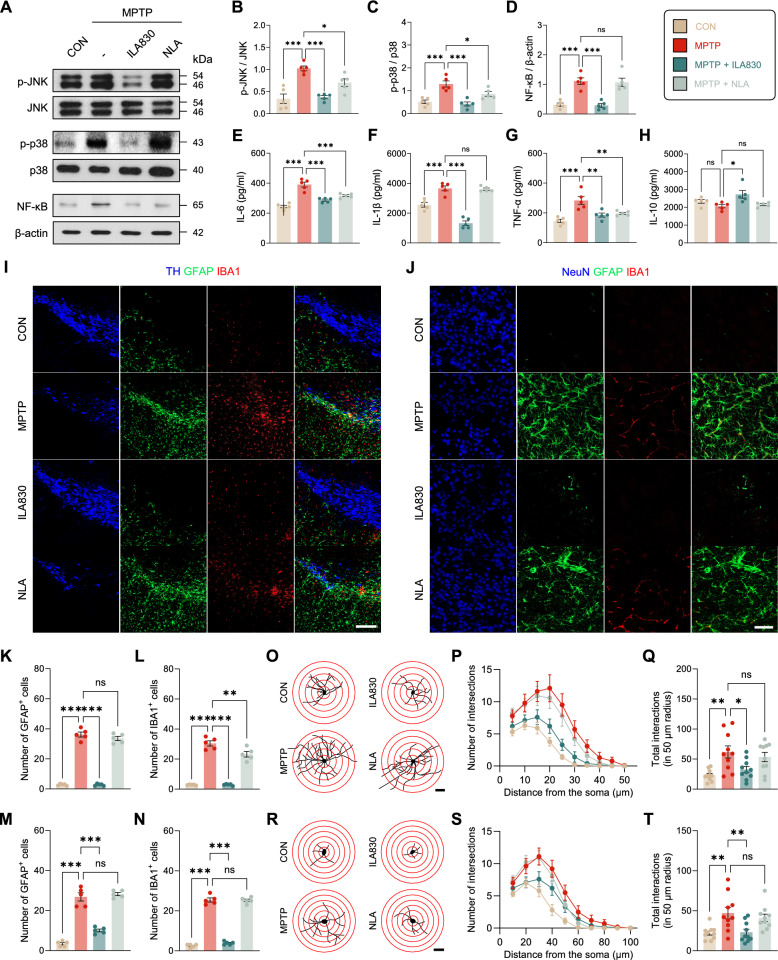
Effects of ILA830 on inflammatory responses and glial activation in the brain. **A** Representative western blot images of JNK, p38, NF-κB, and β-actin in the SN. **B** Statistical analysis of the protein expression levels of p-JNK/JNK in the SN. **C** Statistical analysis of the protein expression levels of p-38/p38 in the SN. **D** Statistical analysis of the ratio of NF-κB, normalized to β-actin in the SN. **E** ELISA analysis of proinflammatory cytokine IL-6 in the ST. **F** ELISA analysis of proinflammatory cytokine IL-1β in the ST. **G** ELISA analysis of proinflammatory cytokine TNF-α in the ST. **H** ELISA analysis of anti-inflammatory cytokine IL-10 in the ST. Representative images of immunofluorescence staining of GFAP- and IBA1-positive cells in the SN and ST. Scale bar = 300 (**I**), 10 (**J**) μm. **K**,**M** Quantitative analysis of GFAP-positive cells in the SN and ST. **L**,**N** Quantitative analysis of IBA1-positive cells in the SN and ST. **O**,**R** Representative images of astrocytic processes via Sholl analysis in the SN and ST. Scale bar = 100 (**I**), 20 (**J**) μm. **P**,**S** The number of intersections of astrocytic processes at different distances from the soma in the SN and ST. **Q**,**T** The number of total intersections within 50 µm radius from the soma in the SN and ST. Data are shown as the mean ± SEM. * *p* < 0.05, ** *p* < 0.01, *** *p* < 0.001

The MAPK pathways play a central role in cell death and survival. In order to evaluate the possible involvement of these pathways in ILA-treated PD mice, we analyzed the phosphorylation state of JNK (Fig. 3A, B) and p38 (Fig. 3A, C) by western blot. The results of the one-way ANOVA demonstrated the statistical significance of the variations among the groups for both JNK [*F*_3, 16_ = 17.63 *p* < 0.001] and p38 [*F*_3, 16_ = 14.98 *p* < 0.001]. Injection of MPTP significantly increased JNK phosphorylation (*p* < 0.001) which was significantly decreased in the ILA830 group (*p* < 0.001). Also, we found that MPTP treatment promoted the phosphorylation of p38 (*p* < 0.001), and ILA played an opposite effect on the phosphorylation of the protein (*p* < 0.001). Mere insertion of needle decreased the phosphorylation levels of JNK (*p* < 0.05) and p38 (*p* < 0.05), albeit the magnitude of the reduction was not as great as with ILA (Fig. 3B, C).

Because the activation of NF-κB signaling pathways is involved in inflammation response, we investigated the effect of ILA on it by western blot (Fig. 3A, D). The results of the one-way ANOVA indicated that there were significant differences among the groups [*F*_3, 16_ = 21.10 *p* < 0.001]. The accumulation levels of NF-κB were increased in MPTP-induced PD mice (*p* < 0.001), and ILA treatment restored the change (*p* < 0.001). These results indicate that ILA at 830 nm alleviates neuroinflammation by regulating MAPKs and NF-κB in MPTP-induced PD mice (Fig. 3D).

Overproduction of inflammatory cytokines, such as IL-6, IL-1β, and TNF-α, results in neurodegeneration. For this purpose, we evaluated the expression of pro-inflammatory cytokines like IL-1β, IL-6, and TNF-α by ELISA (Fig. 3E–G). The results of one-way ANOVA establish the validity of group distinctions for IL-1β [*F*_3, 16_ = 60.92, *p* < 0.001], IL-6 [*F*_3, 16_ = 35.91, *p* < 0.001], and TNF-α [*F*_3, 16_ = 12.48, *p* < 0.001]. Compared to the control group, our data showed a significant upregulation of IL-1β (*p* < 0.001), IL-6 (*p* < 0.001), and TNF-α (*p* < 0.001) in MPTP-intoxicated mice. Acupuncture treatment demonstrated its ability to significantly reduce cytokine levels of IL-6 (*p* < 0.001) and TNF-α (*p* < 0.01) compared to MPTP-injected mice, but only with invasive laser irradiation was the expression level of IL-1β reduced (*p* < 0.001). Additionally, we examined the concentration level of anti-inflammatory cytokine IL-10 to further investigate the inflammatory state (Fig. 3H). One-way ANOVA revealed the significance of group differences [*F*_3, 16_ = 4.855, *p* < 0.05]. Compared to the MPTP group, ILA830 showed a significant increase in its concentration level (*p* < 0.05). These findings suggest that ILA is efficacious in reducing inflammation through the modulation of cytokines.

### ILA alleviates MPTP-induced inflammation by attenuating glial cell activation

Reactive astrogliosis and microglia activation are two mechanisms that contribute to dopaminergic neuronal loss and neuroinflammation in PD. Using immunofluorescence analysis, we studied the expression of GFAP and IBA1, known markers of astrocytosis and microgliosis, respectively (Fig. 3I, J). There were notable distinctions between the groups in the SN [GFAP: *F*_4, 22_ = 111.5, *p* < 0.001, IBA1: *F*_4, 22_ = 87.86, *p* < 0.001] and ST [GFAP: *F*_4, 22_ = 97.02, *p* < 0.001, IBA1: *F*_4, 22_ = 184.9, *p* < 0.001] as shown by the one-way ANOVA results. Our results demonstrated that after MPTP injection, there was a significant increase in both GFAP (*p* < 0.001) and IBA1-positive cells (*p* < 0.001) compared to control mice. ILA significantly reduced the number of both GFAP (*p* < 0.001) and IBA1-positive cells (*p* < 0.001) compared to those that did not receive the treatment (Fig. 3K–N). The same tendency of results was seen both in SN (Fig. 3K, L) and ST (Fig. 3M, N).

The number of process intersections at different radial distances from the neuronal soma was counted using Sholl analysis (Fig. 3O, R). One-way ANOVA revealed that the numbers in each group differed significantly in the SN [*F*_4, 45_ = 4.819, *p* < 0.01] as well as in the ST [*F*_4, 45_ = 4.857, *p* < 0.01]. In the SN, the total intersections in a 50 μm radius were significantly higher in the MPTP group compared to the CON (*p* < 0.01) and ILA830 (*p* < 0.05) groups (Fig. 3P, Q). Moreover, similar results were shown in the ST, where the number of intersections within a 50 μm radius was considerably higher in the MPTP group compared to the CON (*p* < 0.01) and ILA830 (*p* < 0.01) groups (Fig. 3S, T). No significant difference was shown among MPTP, ILA650, and NLA groups in both SN and ST.

### ILA induces clusterings of groups and alterations of metabolites at the site of treatment

**Fig. 4:**
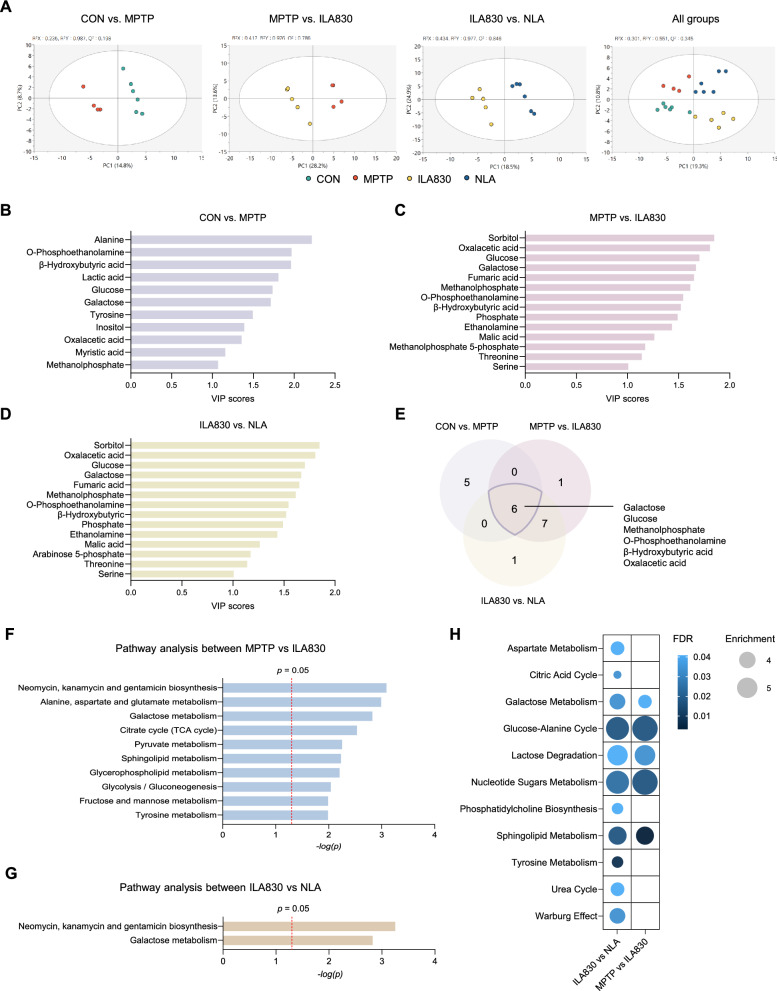
Alterations of metabolites and pathway analysis following ILA treatment in MPTP-induced PD mice. **A** Score plot of PLS-DA. **B**–**D** List of metabolites with VIP scores greater than 1. **E** Venn diagram showing the common metabolites that showed changes in comparisons between groups; galactose, glucose, methanolphosphate, *O*-phosphoethanolamine, β-hydroxybutyric acid, oxalacetic acid. **F**,**G** Bar chart displaying significant pathways. **H** Bubble chart showing significant pathways of **F** and **G**; the size of bubbles represents the enrichment factor of the pathway and the color shows the *p* value of each pathway

After determining the necessity of muscle stimulation and ruling out the possibility of neural transmission, we examined the metabolic changes in the muscle and investigated how they correlate with the treatment outcomes. Muscle samples of the mice legs around acupoint GB34 (*Yangleungchun*) were collected for the purpose of conducting metabolomic investigations, and GC–MS was employed for the analysis. Partial Least Squares Discriminant Analysis (PLS-DA) is a supervised method used for linear discrimination analysis. The PLS-DA results in our study revealed distinct groupings between CON and MPTP (*R*^2^*X*: 0.235, *R*^2^*Y*: 0.987, *Q*^2^: 0.198), MPTP and ILA830 (*R*^2^*X*: 0.417, *R*^2^*Y*: 0.976, *Q*^2^: 0.786), ILA830 and NLA (*R*^2^*X*: 0.434, *R*^2^*Y*: 0.977, *Q*^2^: 0.846), and among all groups (*R*^2^*X*: 0.301, *R*^2^*Y*: 0.551, *Q*^2^: 0.345) (Fig. 4A).

Subsequently, metabolites contributing to the observed distinctions were analyzed. Using Variable Important in Projection (VIP) scores greater than 1.0, significant metabolites were identified in comparisons of CON vs. MPTP (Fig. 4B), MPTP vs. ILA830 (Fig. 4C), and ILA830 vs. NLA (Fig. 4D). Six metabolites—glucose, galactose, methanolphosphate, *O*-phosphoethanolamine, oxalacetic acid, and β-hydroxybutyric acid—were consistently altered across comparisons (Fig. 4E).

### Metabolic pathway enrichment was detected in the muscle of MPTP-induced PD mice via ILA

To explore pathway-level changes, pathway enrichment analysis was conducted using an unpaired Student’s *t* test between MPTP vs. ILA830 and ILA830 vs. NLA. Between MPTP and ILA830, significant pathways included Galactose metabolism, the Citrate cycle (TCA cycle), Alanine, aspartate and glutamate metabolism, and Glycolysis/Gluconeogenesis (Fig. 4F). In contrast, comparisons between ILA830 and NLA revealed Galactose metabolism and related pathways as key differentiators (Fig. 4G). Further enrichment analysis highlighted the glucose-alanine cycle, lactose degradation, and nucleotide sugars metabolism as the most important pathways associated with ILA treatment at 830 nm (Fig. 4H).

### Muscle layers, rather than skin layers or nerve conduction are essential for the effects of ILA830

**Fig. 5:**
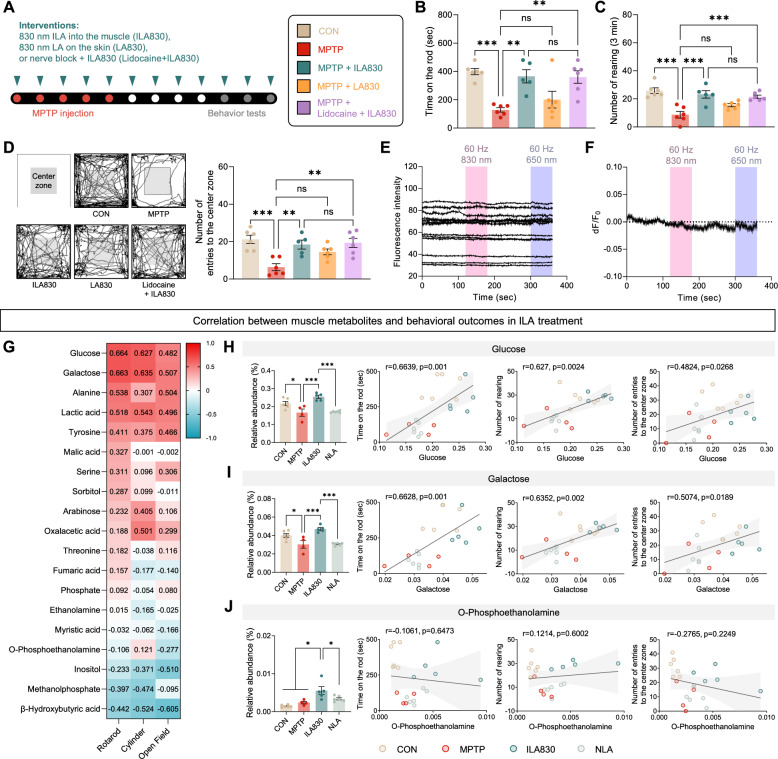
Effects of ILA on motor function, dopaminergic neuronal survival, and their correlation with muscle metabolites in MPTP-induced PD mice. **A** Experimental design. **B** Latency to fall off the rotating rod. **C** Number of rearing in the cylinder test. **D** Number of entries into the center zone recorded in the OF test. **E** Fluorescence raw traces of HT-22 cells expressing GCaMP6s during laser exposure. **F** dF/F0 graph of GCaMP6s-expressing HT-22 cells under laser irradiation at 60 Hz, with wavelengths of 830 nm and 650 nm (830 nm: −0.66 ± 0.29%, 650 nm: −0.81 ± 0.46% *n* = 13 cells). **G** Heatmap demonstrating the correlations of muscle metabolites and behavioral test results. **H**–**J** Bar graphs demonstrating the relative abundance of glucose, galactose, and *O*-phosphoethanolamine, along with their correlation with behavioral test results. Data are shown as the mean ± SEM. * *p* < 0.05, ** *p* < 0.01, *** *p* < 0.001

We investigated the factors that determine the brain’s response to peripheral ILA stimulation. To comprehensively address these objectives, we examined whether muscle stimulation is indispensable. For this experiment we only used 830 nm wavelength which yielded desirable results in the previous experiment. We added two new groups—one that was treated with non-invasive laser, meaning the laser was applied on the surface of the skin, and one that was injected with lidocaine, which is a type of nerve blockers, prior to ILA treatment (Fig. 5A). The experiment initially involved 6 mice in each group. However, one mouse in the ILA830 group died during the MPTP-injecting period, and hence was not accessible for behavioral examinations.

Through one-way ANOVA, the differences among groups in all three behavioral assessments, the rotarod [*F*_4, 24_ = 8.870, *p* < 0.001], cylinder [*F*_4, 24_ = 13.01, *p* < 0.001], and OF test [*F*_4, 24_ = 8.022, *p* < 0.01], were shown to be statistically valid (Fig. 5B–D). In the rotarod test, the reduced time on the rod in the MPTP group (*p* < 0.001) was significantly increased in both ILA830 (*p* < 0.01) and lidocaine + ILA830 (*p* < 0.01) groups. Injection of lidocaine did not statistically affect the results between the ILA groups (*p* > 0.99). The group in which laser was irradiated on the skin surface did not show significance compared to the MPTP group (*p* = 0.70) (Fig. 5B). Similar tendencies were found in the cylinder test, where the decline of rearing in the MPTP group (*p* < 0.001) increased in both groups that were treated with invasive laser, with and without lidocaine pre-treatment (*p* < 0.001) (Fig. 5C). In the OF test, we tested the number of entries to the center zone through which we discovered that the number dropped in the MPTP group (*p* < 0.001) and was restored in both invasive laser treatment groups (*p* < 0.01) (Fig. 5D). In all behavioral tests, the group that was treated with laser on the skin surface did not show statistical difference from the MPTP group, indicating that LA is effective only when applied in the muscle layer. Furthermore, given that the therapeutic effects persisted despite the application of lidocaine to block neuronal activity, it can be concluded that the effects observed in the brain were not mediated by neural transmission.

We further examined the effects of ILA on the neural activity in vitro. Using a neuronal cells with a genetically encoded calcium indicator GCaMP6s, we monitored that the changes of Ca^2+^ fluctuations during laser exposure at the wavelengths of 830 nm or 650 nm, with stimulation frequency of 60 Hz. Results indicated that both laser stimulation did not induce any neural activation, measured by the changes of calcium levels in the neuronal cells (Fig. 5E, F).

### Muscle metabolites that distinguish the groups are correlated with improved motor function in MPTP-induced PD mice

To investigate the relationship between muscle metabolites and behavioral improvements, correlation analysis was performed. Glucose, galactose, alanine, lactic acid, and tyrosine showed considerable positive correlations, and β-hydroxybutyrate was shown to be negatively correlated with the behavioral test results (Fig. 5G).

Among the six metabolites that commonly showed significant difference between CON vs. MPTP, MPTP vs. ILA830, and ILA830 vs. NLA, one-way ANOVA results showed significant differences between groups in glucose [*F*_3, 17_ = 14.2, *p* < 0.001] (Fig. 5H), galactose [*F*_3, 17_ = 14.6, *p* < 0.001] (Fig. 5I), and *O*-phosphoethanolamine [*F*_3, 17_ = 10.2, *p* < 0.001] (Fig. 5J), while methanolphosphate [*F*_3, 17_ = 2.385, *p* = 0.11], oxalacetic acid [*F*_3, 17_ = 2.952, *p* = 0.062], and β-hydroxybutyric acid [*F*_3, 17_ = 1.51, *p* = 0.246] did not show significance between groups. In contrast to the MPTP and NLA groups, the ILA830 group showed a notable increase in glucose (*p* < 0.001) and galactose (*p* < 0.001) levels, restoring to the level of the control group (Fig. 5H, I). On the other hand, the level of *O*-phosphoethanolamine rose only in the ILA830 group (*p* < 0.05) compared to all the other groups (Fig. 5J).

Next, Pearson’s correlation analysis was performed to analyze the correlation between the level of identified metabolites and behavioral test results. Glucose showed significant positive correlation with the rotarod (*r* = 0.6639, *p* < 0.01), cylinder (*r* = 0.6264, *p* < 0.01), and OF (*r* = 0.4824, *p* < 0.01) test results (Fig. 5H). Furthermore, there were notable positive correlations observed between galactose and the rotarod (*r* = 0.6628, *p* < 0.01) and cylinder (*r* = 0.6352, *p* < 0.01), and OF (*r* = 0.5074, *p* < 0.05) tests (Fig. 5I). *O*-phosphoethanolamine did not show significant correlation with the rotarod (*p* = 0.6473), cylinder (*p* = 0.6002), or OF (*p* = 0.2249) tests (Fig. 5J). These results suggest that ILA830 induces improvements in motor function and neuroprotective effects by modulating muscle metabolic pathways.

## Discussion

**Fig. 6:**
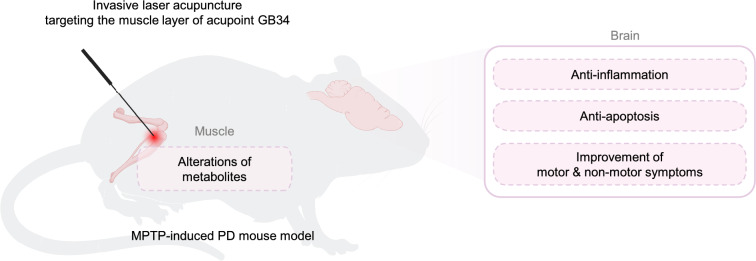
Schematic diagram of ILA830 treatment effects on PD symptom improvement via muscle metabolic alterations and brain recovery in a PD mouse model. ILA at the GB34 acupoint using an 830 nm wavelength stimulates the muscle layer, inducing therapeutic effects in the MPTP-induced PD mouse model. This targeted stimulation leads to significant alterations in muscle metabolites, such as glucose and galactose, enhancing systemic energy availability. These peripheral metabolic changes are proposed to influence the brain, leading to anti-inflammatory and anti-apoptotic effects. Consequently, ILA treatment contributes to the improvement of both motor and non-motor symptoms observed in PD

This study provides compelling evidence that ILA830 significantly ameliorates both motor and non-motor symptoms of PD through the protection of dopaminergic neurons and reduction of neuroinflammation (Fig. 6). To the best of our knowledge, this is the first study to demonstrate that deep muscle layer stimulation of acupoints with laser irradiation can improve both motor and non-motor symptoms of PD. These findings suggest that ILA could be an effective approach for alleviating the symptoms of PD.

PD is characterized by the degeneration of dopaminergic neurons in the SN [27, 28]. The nigrostriatal pathway in the brain plays a crucial role in the synthesis of dopamine, a vital neurotransmitter responsible for regulating balance and movement. Furthermore, TH, a crucial enzyme that controls the rate at which dopamine is produced and released, significantly impacts these processes [29]. MPTP, a compound with a high affinity for lipids, easily crosses the blood–brain barrier (BBB) and specifically damages dopaminergic neurons in the nigrostriatal area, inducing neurochemical and histological changes that resemble PD in primates and rodents [30]. In our research, we used MPTP to model PD, and the motor function assessment revealed that MPTP-treated mice exhibited shorter duration on the rotarod, and a decreased number of rearing behaviors in the cylinder test compared to mice given normal saline. These findings align with prior research indicating motor dysfunction as a result of MPTP injection [31]. However, mice treated with MPTP with ILA showed prolonged latency to fall off the rotarod and elevated rearing rate compared to mice treated only with MPTP. The results were particularly significant in the ILA830 group, which received treatment with ILA at a wavelength of 830 nm, in comparison to the ILA650 and NLA groups. In addition, ILA830 group exhibited increased number of entries to the center zone, indicating improvement of non-motor symptoms as well. Our investigation also found that ILA at a wavelength of 830 nm, not at 650 nm or with non-laser sacupuncture, mitigates the degeneration of dopaminergic neurons in the SN and dopaminergic fibers in the ST of mice with MPTP-induced PD. This suggests that the 830 nm wavelength is particularly effective in providing neuroprotection in the PD model.

The differential effects of PBM depending on the wavelength were also shown in previous studies [32, 33]. They suggest that optimal wavelengths for PBM treatment may differ depending on the specific condition or disease being treated. For instance, while our study demonstrated that ILA at 830 nm has better outcomes in the PD model, a study using the same equipment reported superior results for chronic low back pain with ILA at 650 nm compared to 830 nm [22]. This discrepancy further underscores the condition-specific nature of optimal ILA wavelengths. The mechanisms by which different wavelengths influence biological responses are still being explored. Existing literature suggests that cytochrome C oxidase, a component of the mitochondrial oxidative phosphorylation complex, serves as the primary photoreceptor for shorter wavelengths (600–800 nm). In contrast, longer wavelengths (above 800 nm) might activate light-sensitive ion channels, influencing cellular functions differently [34, 35]. However, the mechanisms underlying the wavelength-dependent effects were not investigated in this study and should be explored in future research. Overall, our findings highlight the potential of ILA830 as a therapeutic approach for PD, emphasizing the importance of selecting optimal wavelength parameters for maximizing behavioral and neuroprotective benefits.

Next, we investigated the molecular mechanisms in the brain that underlie the efficacy of 830 nm wavelength ILA, which has demonstrated significant effects in improving behavioral functions and protecting nigrostriatal dopaminergic neurons in the PD model.

Previous research has indicated that the death of dopaminergic neurons caused by MPTP is associated with excessive production of α-synuclein, leading to oxidative stress and motor impairment [36, 37]. Our study demonstrates that ILA830 therapy effectively suppresses the activation of α-synuclein in the SN and ST caused by MPTP. We also examined the Akt signaling pathway, which is crucial for the survival of dopaminergic neuron [38]. MPTP treatment reduced p-Akt/Akt ratios, but ILA830 inhibited this decrease, indicating its role in promoting cell survival. Activation of Akt promotes cell survival by inhibiting proapoptotic proteins like Bax and upregulating anti-apoptotic proteins like Bcl-2 [39, 40]. Our western blot results revealed that ILA830 treatment effectively counteracted the MPTP-induced decrease in Bcl-2 expression and increase in Bax expression, demonstrating the anti-apoptotic activity of ILA830. Therefore, our research indicates that ILA830 can prevent dopaminergic neuron degeneration by activating the Akt signaling pathway, contributing to the overall neuroprotective effects observed in this PD model.

Neuroinflammation plays a crucial role in driving neurodegeneration in PD. Activated microglia in the SN release proinflammatory cytokines such as TNF-α, IL-1β, and IL-6, which are linked to the onset of PD [41]. Our study shows that ILA830 decreases these proinflammatory cytokines in mice with MPTP-induced parkinsonism while increasing the anti-inflammatory cytokine IL-10. Additionally, inflammatory mediators from glial cells significantly contribute to dopaminergic neuron degeneration [42, 43]. Normally supportive microglia and astrocytes become sources of neurotoxic substances in PD [44, 45]. Our research demonstrates that ILA830 reduces the activation of both microglia and astrocytes in the SN and ST regions, suggesting that inhibiting glial activation reduces neuroinflammation, thereby contributing to the neuroprotective effects of ILA. Furthermore, the MAPK pathway plays a crucial role in the inflammatory response in PD [46]. Elevated levels of phosphorylated JNK and p38, which are associated with neuronal death and inflammation, have been observed in PD patients [47–49]. Our study revealed that ILA830 treatment reversed the MPTP-induced increases in phosphorylated JNK and p38, suggesting that ILA830 mitigates PD via the inhibition of the MAPK signaling pathways, thus reducing neuroinflammation.

As a next step, we sought to understand the peripheral factors that determine the changes in the brain induced by ILA830 stimulation. Initially, as previously mentioned, we found that ILA830 shows better outcomes with the laser on compared to the laser off, establishing the necessity of laser activation for its efficacy. We then investigated which layer, the skin or the muscle, is crucial as the target of laser stimulation. It is known that 830 nm laser irradiation can penetrate to a depth of 2–3 mm [50], but over 80% of the radiation is absorbed by the skin layer [51], resulting in a minimal amount of laser actually reaching the deeper tissue. Therefore, we compared the effects of the conventional non-invasive LA method targeting the skin with our ILA830 method targeting the muscle. The results demonstrated that targeting the muscle with ILA830 significantly improved PD behavioral impairments, indicating the importance of targeting the muscle layer in the effectiveness of ILA830 in the PD model.

In our previous studies, we found that neural transmission plays a crucial role in the improvement of PD symptoms induced by manual acupuncture stimulation [52]. Therefore, we wondered whether neural transmission is also important for the effects of ILA830. Thus, we applied nerve blocks using lidocaine to observe whether neural transmission is also involved in the effects of ILA830. Contrary to our expectations, the nerve block did not inhibit the effects of ILA830. Consequently, we hypothesized that the muscle plays a significant role in mediating the effects of ILA830. To validate this hypothesis, we decided to observe metabolomic changes in the muscle induced by ILA830 and investigate which metabolites are correlated with therapeutic outcomes.

The analysis of muscle metabolomics provides critical insights into the biochemical changes that occur in muscle tissue [53] in response to interventions such as ILA. In the context of PD, where muscle dysfunction often accompanies neurodegeneration, understanding these metabolic changes is essential [54]. Our PLS-DA analysis revealed distinct groupings between different treatment groups, highlighting significant metabolic changes induced by ILA830. The metabolites contributing to these differences were identified, and the analysis of these metabolites revealed that glucose, galactose, and *O*-phosphoethanolamine levels were significantly different between groups, with glucose and galactose levels restored to control levels in the ILA830 group compared to MPTP group. Pearson’s correlation analysis demonstrated positive correlations between glucose and galactose levels with improved motor function in the rotarod and cylinder tests, suggesting their role in mediating the therapeutic effects of ILA830.

Our findings indicate that ILA830 effectively modulates specific metabolic pathways in muscle tissue. The enrichment analysis of metabolic pathways further highlighted significant alterations in pathways such as alanine, aspartate and glutamate metabolism, galactose metabolism, and the citrate cycle. These pathways are crucial for energy production and neurotransmitter synthesis [55–57], which are essential for maintaining motor function and neuroprotection. Our pathway enrichment analysis also revealed that ILA830 treatment significantly impacts the glucose-alanine cycle, lactose degradation, and nucleotide sugars metabolism [58, 59], suggesting the importance of these pathways in the muscle’s response to ILA830. We also found that the levels of glucose and galactose are positively correlated with non-motor function as well as motor function, indicating the importance of elucidated metabolic pathways. These findings provide a deeper understanding of how peripheral stimulation can influence central outcomes, indicating the systemic nature of ILA’s therapeutic effects.

Furthermore, recent evidence suggests that muscle metabolism and glucose utilization may influence central outcomes, such as neuroinflammation and glial activation. ILA830 enhances glucose and galactose metabolism in muscle tissue, improving systemic energy availability and supporting anti-inflammatory pathways [60]. Proper glucose metabolism in the brain reduces neuroinflammation by supporting astrocytes and neurons through the “astrocyte-neuron lactate shuttle,” wherein glucose is converted into lactate by astrocytes and utilized by neurons as an energy source, mitigating oxidative stress [61]. Additionally, enhanced glucose metabolism strengthens BBB integrity, reducing the entry of inflammatory mediators and decreasing glial activation [62, 63]. These mechanisms likely contribute to the reduction in neuroinflammation and dopaminergic neuron protection observed in our study.

Overall, the metabolomic analysis of muscle tissue shows the importance of peripheral metabolic changes in mediating the effects of ILA830. By identifying key metabolites and pathways involved in the response to ILA, we can better understand the mechanisms underlying its neuroprotective and therapeutic effects. This knowledge can guide the development of more targeted and effective treatments for PD, emphasizing the significance of selecting optimal wavelength parameters of ILA for maximizing both behavioral and neuroprotective benefits.

This study represents a significant advancement in the exploration of therapeutic strategies for PD, as it identifies optimal wavelengths and targets for ILA. To the best of our knowledge, it is the first study to analyze the muscle metabolome in a PD model, providing novel insights into the metabolic changes associated with ILA treatment and PD. Additionally, we have demonstrated that ILA operates through a distinct mechanism compared to conventional acupuncture, which primarily exerts its effects through neural mediation. These findings suggest that ILA could offer a unique and potentially more effective therapeutic approach for PD.

However, there are several limitations to our study that must be acknowledged. Firstly, the experiments were conducted with a relatively small number of animals, which may limit the generalizability of the results. Future studies should include larger sample sizes to validate and extend our findings. Secondly, while we observed significant therapeutic effects of ILA, the specific mechanisms by which laser stimulation of the legs impacts the brain and alleviates PD symptoms remain unclear. This indicates a need for further investigation to elucidate the pathways involved in this novel treatment. Additionally, the long-term effects and safety of ILA require comprehensive evaluation before it can be considered for clinical application.

This study provides evidence that ILA improves motor functions and protects dopaminergic neurons in MPTP-induced PD mice exclusively at the 830 nm wavelength, specifically when it is irradiated in the muscle layer. The treatment also reduces neuroinflammation and induces beneficial muscle metabolomic changes, such as increased glucose and galactose levels, which correlate with motor and non-motor improvements. These findings suggest that ILA830 is a promising therapeutic approach for PD. Future research should focus on elucidating the mechanisms of wavelength-dependent effects and optimizing ILA for clinical applications.

## Data Availability

The data that support the findings of this study are available from the corresponding author upon request.
